# Comparison of the receptor FGFRL1 from sea urchins and humans illustrates evolution of a zinc binding motif in the intracellular domain

**DOI:** 10.1186/1471-2091-10-33

**Published:** 2009-12-18

**Authors:** Lei Zhuang, Andrei V Karotki, Philip Bruecker, Beat Trueb

**Affiliations:** 1 Department of Clinical Research, University of Bern, 3010 Bern, Switzerland; 2 Department of Biochemistry, University of Zürich, 8057 Zürich, Switzerland; 3 Living Elements Ltd., Vancouver BC V6J 1M7, Canada; 4 Department of Rheumatology, University Hospital, 3010 Bern, Switzerland

## Abstract

**Background:**

FGFRL1, the gene for the fifth member of the fibroblast growth factor receptor (FGFR) family, is found in all vertebrates from fish to man and in the cephalochordate amphioxus. Since it does not occur in more distantly related invertebrates such as insects and nematodes, we have speculated that FGFRL1 might have evolved just before branching of the vertebrate lineage from the other invertebrates (Beyeler and Trueb, 2006).

**Results:**

We identified the gene for FGFRL1 also in the sea urchin Strongylocentrotus purpuratus and cloned its mRNA. The deduced amino acid sequence shares 62% sequence similarity with the human protein and shows conservation of all disulfides and N-linked carbohydrate attachment sites. Similar to the human protein, the S. purpuratus protein contains a histidine-rich motif at the C-terminus, but this motif is much shorter than the human counterpart. To analyze the function of the novel motif, recombinant fusion proteins were prepared in a bacterial expression system. The human fusion protein bound to nickel and zinc affinity columns, whereas the sea urchin protein barely interacted with such columns. Direct determination of metal ions by atomic absorption revealed 2.6 mole zinc/mole protein for human FGFRL1 and 1.7 mole zinc/mole protein for sea urchin FGFRL1.

**Conclusion:**

The FGFRL1 gene has evolved much earlier than previously assumed. A comparison of the intracellular domain between sea urchin and human FGFRL1 provides interesting insights into the shaping of a novel zinc binding domain.

## Background

FGFRL1 is the fifth member of the fibroblast growth factor receptor (FGFR) family. It was originally discovered in a cDNA library prepared from human cartilage [1], but it is also expressed at relatively high levels in bone and some muscles. Furthermore, all tissues examined so far contain low, basal levels of FGFRL1 [1-4].

The classical FGFRs (FGFR1-FGFR4) are cell surface proteins with a single transmembrane domain, three extracellular Ig-like loops and an intracellular protein tyrosine kinase domain [5-7]. The first Ig-loop is separated from the second by a stretch of negatively charged amino acids that are sometimes referred to as "acidic box". The classical receptors are widely expressed in mammalian tissues and control a diversity of biological functions, including proliferation, migration and differentiation of many cell types. Germline mutations in FGFR genes are able to cause a number of skeletal disorders such as craniosynostosis syndromes and chondrodysplasias [8]. Somatic mutations in FGFRs can lead to unrestricted cellular growth and cancer as observed in bladder carcinomas, multiple myelomas and chronic myeloproliferative diseases [9].

The function of the fifth FGFR is not yet understood in detail. Similar to the classical FGFRs, it contains three extracellular Ig-like loops and a transmembrane domain [1-3]. However, in contrast to the other FGFRs, it lacks the intracellular protein tyrosine kinase domain that would be required for signal transduction by trans-phosphorylation. Instead, it contains an intracellular domain with a peculiar histidine-rich motif that does not share much homology with any known protein.

Recombinant FGFRL1 interacts with heparin and FGF2 in a manner analogous to the classical FGFRs [10]. When overexpressed in MG63 osteosarcoma cells, it has a negative effect on cell proliferation [10]. In a luciferase reporter gene experiment, it is capable of inhibiting the activity of the FGF inducible responsive promoter element FIRE [11]. Furthermore, its synthesis is significantly up-regulated during differentiation of myoblasts into myofibers [12]. We have therefore concluded that FGFRL1 might function as a decoy receptor that binds FGF ligands and sequesters them from the other FGFRs. In this way, it might inhibit cell proliferation and promote cell differentiation.

More information about the function of the novel receptor can be obtained from animal experiments. When the synthesis of FGFRL1 was down-regulated with morpholino constructs in a zebrafish model [13], the animals failed to properly form the pharyngeal arches. It is therefore likely that FGFRL1 is involved in the development of the gill cartilage. Recently we generated mice with a targeted disruption of the FGFRL1 gene [12]. These knock-out mice develop normally to term, but die immediately after birth due to respiratory distress. The respiratory problems are explained by a severely reduced diaphragm muscle that is not strong enough to inflate the lungs after birth. Another research group that has generated similar FGFRL1 deficient mice reported on alterations in the skeleton and the heart, in addition to the malformed diaphragm [14]. The involvement of FGFRL1 in the formation of the skeleton is in accordance with the identification of the first human mutation in a patient who suffers from Antley Bixler Syndrome [11]. This patient displays a frameshift mutation in the last exon of the FGFRL1 gene and shows craniosynostosis, radio-ulnar synostosis and genital abnormalities. As demonstrated by cell culture experiments, the mutant protein stays for a prolonged period of time at the plasma membrane, where it interacts with FGF ligands, while the wild-type protein is rapidly removed from the plasma membrane and sorted to lysosomes [11]. Taken together, these studies suggest that FGFRL1 controls the proper development of the musculoskeletal system.

The gene for FGFRL1 is found in all vertebrates from fish to man [1-3,10,15,16]. Teleostean fish have even two genes, fgfrl1a and fgfrl1b, because they have undergone a whole genome duplication [17]. Vertebrates represent one subphylum of the chordates, together with the cephalochordates and the urochordates. Recently, we were able to identify the gene for FGFRL1 also in the cephalochordate Branchiostoma floridae, but not in the urochordate Ciona intestinalis [18]. We therefore concluded that it might have evolved just before branching of the vertebrate lineage from the other chordates.

Here we cloned a cDNA for FGFRL1 from the sea urchin Strongylocentrotus purpuratus. The fact that sea urchins also possess the gene for FGFRL1 demonstrates that this gene must be much older than originally thought.

## Results

### An FGFRL1 related gene from Ciona intestinalis

A search through the completed genome sequence of the sea squirt Ciona intestinalis allowed the identification of a newly annotated gene (ID 100185880) that shows striking similarity to human FGFRL1. The corresponding mRNA (XM_002125800.1) encodes a protein (XP_002125836.1) of 658 amino acid residues with three typical Ig-like domains, a transmembrane domain and an intracellular domain. This putative protein shares 35%-36% sequence identity (45% sequence similarity if conservative amino acid substitutions are included) with FGFRL1 from humans and lancelet. The similarity is confined to the three Ig-like domains, which show 45%-49% sequence similarity with the three Ig-like domains from vertebrates and lancelet (Table 1). The other parts of the putative protein do not share much similarity. In particular, the N-terminal domain of the C. intestinalis protein is roughly 100 amino acid residues longer than the vertebrate proteins and cannot be aligned. The intracellular, C-terminal region is also longer than the vertebrate counterpart and shows less than 20% sequence identity with the human protein. No motif related to the histidine-rich sequence of vertebrate FGFRL1 can be identified at the C-terminal end of the Ciona protein. Curiously enough, the putative protein also lacks a signal peptide that would be required for insertion of the receptor into the plasma membrane. Nevertheless, the trace archive of the National Center for Biotechnology Information (NCBI) contains 17 expressed sequence tags (ESTs) from C. intestinalis that partially overlap with the entire length of the predicted mRNA, thus confirming sequence and authenticity of the predicted protein.

**Table 1:** Protein similarities

	Human	Lancelet	Sea Urchin	Mouse	Chicken	Frog	Fish A	Fish B	Sea Squirt
**Human**	100	72	62	96	86	83	77	77	49

**Lancelet**		100	61	73	73	72	69	69	47

**Sea Urchin**			100	63	62	64	61	61	45

**Mouse**				100	85	83	78	77	48

**Chicken**					100	91	78	79	48

**Frog**						100	78	80	48

**Fish A**							100	83	46

**Fish B**								100	46

**Sea Squirt**									100

**Figure 1:**
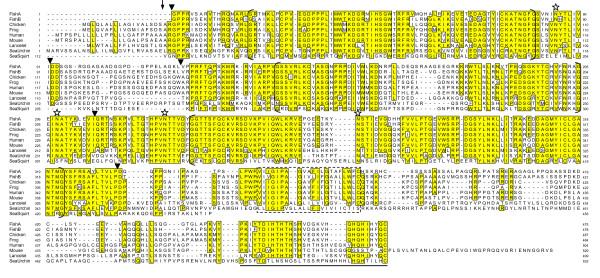
**Alignment of the FGFRL1 sequences from eight different species**. Identical residues are boxed. The putative signal peptidase cleavage site is shown by an arrow. The three Ig-like domains are marked by brackets. The transmembrane domain is given by a stippled box. Conserved glycosylation sites (NXT) are indicated by asterisks. The relative positions of introns in the corresponding genes are shown by triangles. The sequence that was used for the preparation of the zinc-binding GST fusion protein is underlined. The accession numbers are: FishA (Takifugu rubripes A) BN000669, FishB (Takifugu rubripes B) BN000670, Chicken AJ535114, Frog (Silurana tropicalis) AJ616852, Human AJ277437, Mouse AJ293947, Lancelet (Branchiostoma floridae) AJ888866, Sea Urchin (Strongylocentrotus purpuratus) FN252817, Sea Squirt (Ciona intestinalis) XP_002125836. From the sea squirt sequence only the conserved domain (residues 112-456) is included.

The three Ig-like domains were aligned with the FGFRL1 sequences from five vertebrates and lancelet (Fig. 1). In the resulting multiple sequence alignment, all the cysteine residues that participate in disulfide-bond formation of the three Ig-like loops are conserved. In contrast, the attachment sites for asparagine-linked carbohydrates are not conserved. The C. intestinalis sequence contains a total of 7 glycosylation signals (NXT/S) from which only two match glycosylation sites in the vertebrate sequences.

**Figure 2:**
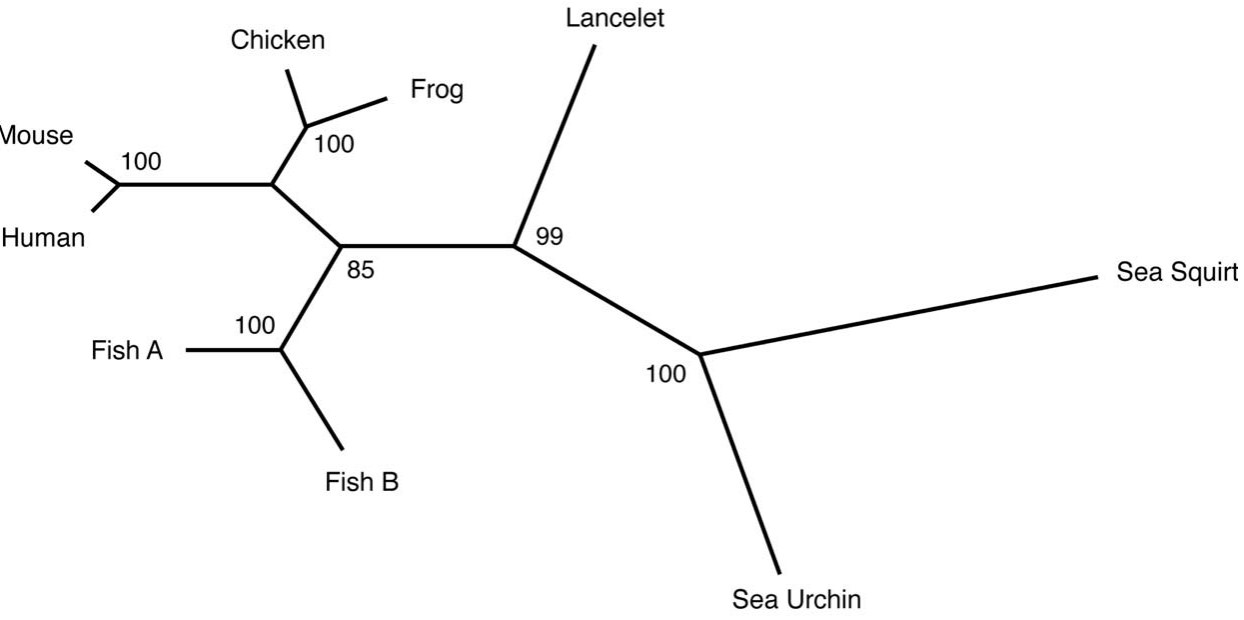
**Phylogenetic analysis of the FGFRL1 sequences**. An unrooted tree was built by the neighbour joining method. Bootstrap values from 1000 random replicates are indicated at the nodes. The length of the branches inversely correlates with the degree of similarity. Only the sequences from the extracellular domains without signal peptides and transmembrane domains were used.

The conserved part of the sea squirt sequence with the three Ig-like domains was used to build a phylogenetic tree by the neighbour joining method (Fig. 2). In the resulting unrooted tree, the Ciona sequence is placed on one branch together with the lancelet sequence as would be expected for two members of the chordata. However, the relative distance between the sea squirt and lancelet is extremely large such that the sea squirt behaves like an outgroup.

When the predicted mRNA sequence was compared to the genome sequence of C. intestinalis, 11 coding exons could be identified (not shown). An inspection of the splice phases showed that in 6 cases the exon/intron boundaries disrupt the codons for the amino acids after the first nucleotide (splice phase 1), in 3 cases after the second nucleotide (splice phase 2) and in one case after the third nucleotide (splice phase 0). This gene structure differs completely from that of the human FGFRL1 gene which contains only 6 coding exons (and a 5' non-coding exon) and in which the splice boundaries disrupt the amino acid codons always after the first nucleotide (splice phase 1).

It is difficult to speculate on the relationship of the Ciona gene with the vertebrate FGFRL1 genes. The striking conservation of the three Ig-like domains suggests that the Ciona gene is the orthologue of human FGFRL1. However, the substantial differences in the gene structure, the lack of the histidine-rich domain at the C-terminus and the apparent absence of a signal peptide argue against this possibility. One way to explain the controversial findings would be the assumption that the predicted C. intestinalis gene still contains many sequencing errors. However, the existence of 17 overlapping EST clones argues against this possibility. Another way to reconcile the findings would be that the Ciona gene does no longer serve an active function and consequently is about to evolve or decay at an accelerated speed.

### Cloning of sea urchin FGFRL1

A similar search through the recently published genome sequence of the sea urchin Strongylocentrotus purpuratus (assembly Spur V2.1 [19]) revealed one gene (LOC578697) that is highly homologous to human FGFRL1. The predicted mRNA sequence has a length of 1674 nt and codes for a protein of 557 amino acids sharing 48% sequence identity (55% similarity) with human FGFRL1. However, a detailed comparison of the predicted mRNA sequence with the S. purpuratus genome indicates that several portions must have been misinterpreted by automated gene analysis with the program Gnomon. First, the N-terminus of the predicted protein does not include a hydrophobic signal peptide required for insertion of the receptor into the plasma membrane. Secondly, the sequence corresponding to the linker between Ig domain I and II (acidic box) is encoded by many short fragments that repeatedly change the splicing phase.

We therefore tried to verify the predicted mRNA sequence by comparing it with EST clones. Although the Trace Archive of NCBI contains numerous EST clones from S. purpuratus, none of them overlaps with the predicted sequence. EST databases from related species were therefore consulted. In a database compiled at the Max Planck Institute for Molecular Genetics in Berlin (http://www.molgen.mpg.de/~ag_seaurchin/[20]) we finally found two EST clones from the related sea urchin Paracentrotus lividus that are highly homologous to human FGFRL1. One covers the sequence corresponding to human amino acid residues 14-212 and shows 47% identity at the amino acid sequence level, the other covers the sequence corresponding to residues 14-69 and shows 51% identity.

In order to establish the correct sequence for S. purpuratus FGFRL1, we set out to clone the corresponding mRNA. Total RNA was isolated from a freshly collected specimen of S. purpuratus and transcribed into first strand cDNA. Primers were designed according to the 5' sequence from the P. lividus EST clones and the 3' sequence from the predicted S. purpuratus mRNA. With the help of these primers, we were able to amplify a cDNA fragment of 1.6 kb. Direct sequencing of this fragment revealed an open reading frame of 1599 bp (accession number FN252817) that displayed 50% sequence identity with the human mRNA sequence. The open reading frame could be translated into an amino acid sequence of 532 residues, which showed 48% sequence identity (55% sequence similarity) with human FGFRL1.

The corrected mRNA sequence for S. purpuratus FGFRL1 contained 11 ambiguous nucleotides. It is likely that these sites represent single nucleotide polymorphisms (SNPs) since none of them caused a change in the derived amino acid sequence and since they persisted when the cloning/sequencing experiment was repeated. Since the RNA had been isolated from a single animal the SNPs must represent two haplotypes originating from the two different alleles. Indeed, when the PCR product was subcloned into the sequencing vector pUC and resequenced from a single clone, no ambiguous nucleotides were observed. High heterozygosity has previously been noted during the elucidation of the S. purpuratus genome [19].

### Gene structure

When the mRNA sequence was aligned with the S. purpuratus genome, six exons could be identified (Table 2). All the splice sites of these exons conform to the consensus rules for donor (GTRNRT) and acceptor (YAG) sequences. The splice phases and the exact splice sites are fully conserved between the sea urchin and the human gene [1]. Exon 1 codes for the signal peptide, exon 2 for Ig domain I, exon 3 for the linker region (acidic box), exon 4 for Ig domain II, exon 5 for Ig domain III and exon 6 for the transmembrane segment and the intracellular domain as previously observed in the human gene.

**Table 2:** Structure of the Fgfrl1 gene from Strongylocentrotus purpuratus

**Exon**	**Acceptor**		**Donor**	**Splice phase**	**Exon size**	**Domain encoded**
1	cgccgataga	ATGGCTCGGG...	...GAACTTCGAGgtaagt	1	>85 bp	5'UTR, signal
		MetAlaArgV	GluLeuArgG			
2	gtcattgcag	GGCCACCAAA...	...ACAATTACTGgtgagt	1	273 bp	Ig I
		lyProProLy	ThrIleThrA			
3	cttaccatag	ATGGATCCAA...	...ACATCAGGAAgtaagt	1	84 bp	acidic box
		spGlySerSe	ThrSerGlyT			
4	cttaccatag	CTATGCCTCA...	...GATGTAGTAGgtgagt	1	282 bp	Ig II
		hrMetProGl	AspValValA			
5	gtttatttag	CTATGCCTCA...	...AGATGTAGTAgtgagt	1	281 bp	Ig III
		spGlnValLy	ValPheProA			
6	tcccctatag	ATCCTAACAT...	...TCTTCATTGCtagcat	1	>521 bp	transmembrane,
		spProAsnMe	eLeuHisCys			intracellular, 3'UTR

### Homology to mammalian proteins

When the amino acid sequence of FGFRL1 from S. purpuratus was aligned with the FGFRL1 sequences from vertebrates and lancelet, several regions with high homology could be observed (Fig. 1). Yet, the degree of sequence conservation is not uniformly distributed over the total length of the aligned sequences. Particularly good conservation (>60%) is noted for all three Ig domains, whereas the signal peptide, the linker between Ig domain I and II (acidic box) and the majority of the intracellular domain show negligible conservation (~20%). The location of the six cysteines that are involved in disulfide bond formation is fully conserved, as is the location of the glycosylation signals NXT for asparagine-bound carbohydrates. Yet, the FGFRL1 sequence from S. purpuratus contains an additional glycosylation site (NSS) at position 169.

The sequence of the extracellular domain (without signal peptide and without transmembrane domain) shares 61-64% identity with FGFRL1 from lancelet and vertebrates (Table 1). This portion of the FGFRL1 sequence was utilized to build a phylogenetic tree by the neighbour joining method (Fig. 2). In the resulting unrooted tree, FGFRL1 from sea urchin, sea squirt and lancelet are placed on one branch, FGFRL1 from fish on a second branch and FGFRL1 from mammals, chicken and frog on a third. The confidence for placement of the individual nods as determined by the bootstrapping method was found to be highly significant (85%-100%). This phylogenetic tree is in good agreement with our current understanding of vertebrate evolution with the only exception of the sequence from C. intestinalis as already discussed above.

### Tissue expression of sea urchin FGFRL1

**Figure 3:**
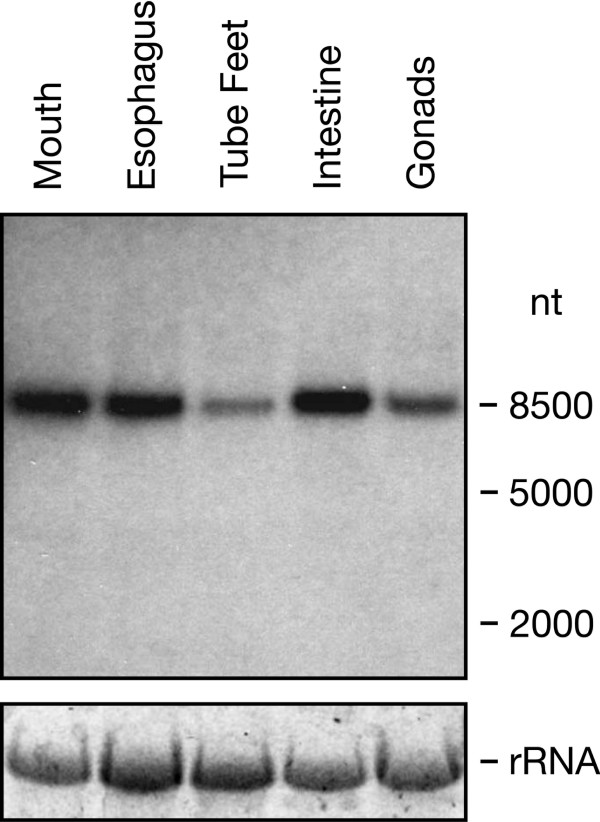
**Expression of the FGFRL1 gene in different tissues from S. purpuratus**. A radioactively labelled cDNA probe corresponding to the sequence for amino acid residues 1-374 was hybridized to a Northern blot containing 7.5 μg RNA from five different tissues as indicated. The 26S ribosomal RNA stained with ethidium bromide is included as a loading control.

To confirm expression of the FGFRL1 gene in S. purpuratus, we prepared a Northern blot using RNA from five different tissues and the cloned cDNA as a probe. A single band was observed on the resulting blot that migrated with a relative mobility corresponding to 8500 nucleotides (Fig. 3). This size suggests that the open reading frame (1599 nt) is followed by an extremely long 3' untranslated region (6000 nt). Thus, the size of the S. purpuratus mRNA is considerably longer than that of the mouse (2500 nt [15]), human (3000 nt [1]) and chicken (6000 nt[10]) mRNA.

The bands on the Northern blot demonstrate that sea urchin FGFRL1 is expressed in all the five tissues examined, with particularly high levels found in mouth, esophagus and intestine (Fig. 3). This is in sharp contrast to our previous findings with human [1] and mouse [4] tissues, where high level expression seemed to be restricted to cartilage, bone and some muscles.

### Zinc binding

The only region of the intracellular domain that is conserved between FGFRL1 from mammals and S. purpuratus is the histidine-rich motif at the very C-terminal end. In this region, the human sequence contains 10 histidines within the last 26 residues, whereas the S. purpuratus sequence contains 6 histidines within the last 12 residues (Fig. 1). Histidine-rich sequences are usually found in zinc finger proteins [21-23] that are involved in protein-protein interactions and in DNA binding. It is therefore possible that zinc ions bind to the C-terminal region of FGFRL1. Since the sea urchin protein contains a significantly lower number of histidine residues, it might also bind less zinc ions than the human protein.

**Figure 4:**
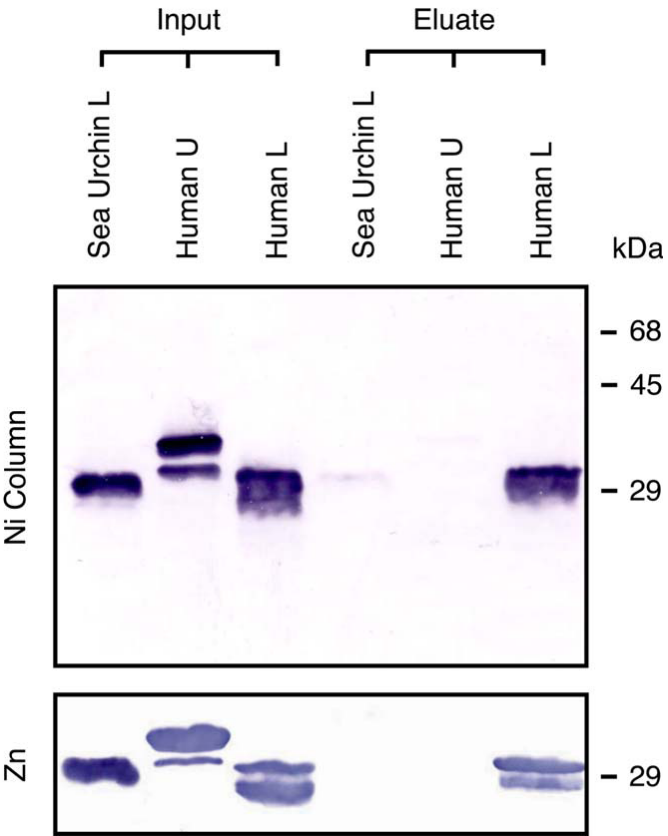
**Interaction of the C-terminal domain from FGFRL1 with zinc and nickel ions**. GST fusion proteins were mixed with nickel or zinc beads, washed and eluted with 1.2 M imidazole. The eluted proteins were resolved on a polyacrylamide gel, transferred to a nitrocellulose membrane and stained with antibodies against GST. Specifically eluted proteins are compared to the starting material (Input). The fusion proteins comprised amino acid residues 400-471 (Human U), 472-504 (Human L) and 492-532 (Sea Urchin L).

To address this question, we expressed three fragments from the intracellular domain of FGFRL1 as GST-fusion proteins in a bacterial expression system, namely an upper human fragment of 72 residues covering amino acids 400-471, a lower human fragment of 33 residues covering amino acids 472-504 and a sea urchin fragment of 41 residues covering amino acids 492-532. On a polyacrylamide gel, the purified fusion proteins migrated with electrophoretic mobilities that are consistent with the calculated molecular weights (Fig. 4). To demonstrate an interaction with metal ions, the fragments were loaded onto nickel columns, washed with loading buffer and eluted specifically with imidazole. Under our experimental conditions, the lower human fragment bound specifically to the column, whereas the upper human fragment as well as the sea urchin fragment barely interacted with the column matrix (Fig. 4). The binding experiments were repeated with a column matrix for which the nickel ions had been exchanged by zinc ions. Again, the lower human fragment bound to the zinc column and could specifically be eluted with 1.2 M imidazole, while the sea urchin fragment as well as the upper human fragment did not bind to the column. These results suggest that the sea urchin protein has considerably lower affinity for zinc ions than the human homologue.

**Figure 5:**
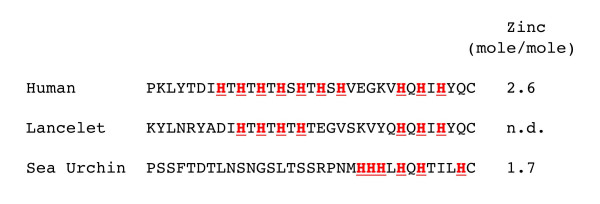
**Comparison of the C-terminal FGFRL1 sequences from humans, lancelet and sea urchin**. Histidine residues are highlighted in bold. Atomic absorption spectroscopy showed that the human protein bound 2.6 mole zinc/mole protein, while the sea urchin protein bound 1.7 mole zinc/mole protein. The standard deviation from three different measurements was < 2%. The zinc content of the lancelet protein was not determined (n.d.), but this sequence was included for reasons of comparison.

The actual amount of zinc ions bound to the expressed fusion proteins was determined by atomic absorption spectroscopy. We found that the upper human fragment contained negligible amounts of zinc (<0.5 mole zinc/mole protein). The lower human fragment bound 2.6 mole zinc/mole protein, the corresponding sea urchin protein 1.7 mole zinc/mole protein (Fig. 5). Taking into account that a small proportion of the recombinant polypeptides might be present in partially degraded or incorrectly folded form, these numbers may correspond to 3 zinc ions for the human and 2 zinc ions for the sea urchin protein, respectively. Neither of the three fragments bound significant amounts of copper ions. Thus, the C-terminus of FGFRL1 interacts specifically with zinc ions and the number of bound zinc ions is higher in the human than in the sea urchin FGFRL1.

## Discussion

FGFRL1 is a transmembrane receptor of the FGFR family. We have previously demonstrated that the FGFRL1 gene occurs in all vertebrates [1,10,15,17], while invertebrates such as insects and nematodes do not appear to possess a related gene. Vertebrates belong to the chordates, a phylum that also includes the subphyla of the cephalochordates and urochordates (tunicates). Recently, we also found the FGFRL1 gene in the cephalochordate Branchiostoma floridae, but not in the urochordate Ciona intestinalis or in more distantly related invertebrates [18]. We therefore concluded that the FGFRL1 gene might have evolved just before branching of the vertebrate lineage from the other chordates.

This conclusion appears to be wrong. The recently sequenced genome of the sea urchin S. purpuratus indicates that echinoderms are the closest known relatives of the chordates [19]. Here we were able to clone a cDNA for FGFRL1 from S. purpuratus and show that it is closely related to the vertebrate homologue. The sea urchin protein shares 61-64% amino acid sequence identity with the vertebrate protein and displays the same domain structure with all disulfides and asparagine-linked carbohydrates conserved. A related gene could also be identified in the genome of the urochordate C. intestinalis. However, the function of this gene remains questionable at the moment since the encoded protein lacks the signal peptide required for insertion into the plasma membrane. At any rate, the presence of FGFRL1 in sea urchins demonstrates that the FGFRL1 gene is much older than previously assumed.

Not only the protein sequence but also the gene structure is fully conserved between sea urchins and vertebrates. In either case there are six protein coding exons and the splice phases and splice sites are preserved. The three exons encoding the three Ig domains have correctly been predicted by automated annotation of the S. purpuratus genome [19]. However, the first and the third exon have been misinterpreted by the automated gene prediction software. The first exon codes for the signal peptide that, apart from its hydrophobic character, does not show much conservation among different species. In the human and rodent genome, this exon is found close to the promoter region, approximately 10 kbp upstream from the rest of the gene [1,15]. The third exon codes for the linker between Ig loop I and II. This exon has probably been missed by automated prediction because its derived amino acid sequence displays a particularly low degree of conservation between sea urchins and vertebrates (<20%). This region might serve as a flexible linker in the FGFRL1 protein that allows the first Ig domain to fold back to the second and third Ig domains [6,7]. In the case of the classical receptor FGFR1, this linker has been termed "acidic box" because it contains a total of 13 aspartic and glutamic acids within 28 residues. When it folds back, Ig domain I may shield the sites for interaction with heparin and FGF ligands. The acidic box might therefore be involved in the modulation and regulation of heparin and FGF binding. Moreover, the acidic box has been implicated in the interaction of FGFR1 with the adhesion molecule NCAM [24]. However, the corresponding region of human FGFRL1 contains only 6 acidic amino acids within 27 residues. It is therefore not clear whether it might serve a similar function to the acidic box of FGFR1.

In contrast to the extracellular domain with the three highly conserved Ig loops, the intracellular domain displays a very low degree of conservation between sea urchins and humans (23%). The only region of this domain that reveals some similarity is the histidine-rich motif at the very C-terminal end. Here FGFRL1 from humans and sea urchins contain nearly 50% histidines (10 histidines within 26 residues for humans, 6 histidines within 12 residues for S. purpuratus). In the human sequence, the histidines alternate with threonine, serine or glutamine residues, whereas in the sea urchin sequence, three of the six histidines occur in a row without interspersed residues. Histidine-rich sequences typically occur in zinc finger domains that form interfaces for protein-protein and protein-DNA interactions [21-23]. Such zinc fingers are found in cytoskeletal and focal adhesion proteins (e.g. zyxin) but also in nuclear proteins and transcription factors (e.g. homeobox proteins). Most zinc fingers conform to the sequence C2H2, where one zinc ion is coordinated by two cysteines and two histidines [21]. The sequence of FGFRL1 clearly differs from the classic C2H2 motif as it contains mainly histidines distributed over 12-26 residues. By column chromatography and by atomic absorption spectroscopy we could demonstrate that this region of the human as well as the sea urchin protein interacts with zinc ions. Under our experimental conditions, the C-terminal tail of the human protein bound 3 zinc ions, while that of the S. purpuratus protein bound only 2 zinc ions. The difference in binding capacity could be confirmed by zinc-column chromatography, where the human protein interacted strongly with the column, while the S. purpuratus protein barely interacted at all. Thus, the C-terminal domain of FGFRL1 contains a novel zinc binding motif, which has gradually improved its zinc binding activity during evolution. When we compare FGFRL1 from sea urchins and humans we may therefore observe the appearance and shaping of a new functional domain.

What is the function of this histidine-rich domain? As mentioned above, histidine-rich domains are often found in cytoskeletal proteins where they enable protein-protein interactions [21-23]. There is indirect evidence that the histidine-rich tail might in fact be involved in the interaction of FGFRL1 with cytoskeletal proteins. When full-length FGFRL1 was overexpressed in HEK293 cells and then subjected to subcellular fractionation utilizing a commercial kit, the majority of the proteins was found in the insoluble fraction that is known to contain cytoskeletal proteins [[11]; Rieckmann and Trueb, unpublished results]. In contrast, FGFRL1 lacking the histidine-rich region was primarily found in the Triton X-100 soluble fraction that contains most of the membrane proteins. Unfortunately, the complete insolubility of the full-length protein has so far hampered all our efforts to characterize the putative cytoskeletal interaction partner(s) of FGFRL1.

Based on the zinc content of the human and the sea urchin protein, we concluded above that FGFRL1 must have increased its zinc binding properties during evolution. It is possible that along with the increased zinc-binding properties, the affinity for cytoskeletal proteins may also have improved. In this way, FGFRL1 could gradually have acquired a novel function.

In addition to the lower zinc binding capacity of the S. purpuratus protein, we have also noted another striking difference between human and sea urchin FGFRL1. In S. purpuratus, FGFRL1 is expressed at relatively high levels in all tissues examined, including mouth, esophagus, intestine, gonads and tube feet. In humans and mice, FGFRL1 is primarily expressed in cartilage, bones and some muscles [1,4], while all other tissues express only basal levels of FGFRL1. In this context, it is of interest to note that sea urchins do not possess any cartilage and bone. The particular function that FGFRL1 might play in cartilage and bone must therefore have been acquired after evolution of echinoderms, i.e. FGFRL1 must have taken over additional functions during evolution. A similar observation has previously been made with teleostean fish. Bony fish, including pufferfish and zebrafish, possess two genes for FGFRL1, fgfrl1a and fgfrl1b, because they have undergone a whole genome duplication [17]. While most of the duplicated genes were lost again during evolution, some selected genes were preserved in the genome because they adopted new functions. Notably, the two copies of FGFRL1 have been preserved in the fish genome and today they display slightly different expression patterns. Subfunctionalization might have played a decisive role in maintaining the two FGFRL1 copies during evolution of bony fish.

## Conclusion

The origin of the FGFRL1 gene is much older than previously assumed. Its ubiquitous expression in sea urchins, but its relatively restricted expression in vertebrates lend further support to the notion that FGFRL1 has gradually taken over specific functions during evolution. This process of subfunctionalization is reflected by improvements of its zinc binding capacity. We might therefore witness the shaping of a novel functional domain when we compare the C-terminal end of FGFRL1 from sea urchins and vertebrates.

## Methods

### Cloning of sea urchin FGFRL1

Sea urchins of the species Strongylocentrotus purpuratus were collected from the Pacific Northwest by Living Elements Ltd. The animals were kept in sea water at 12°C with an artificial current. Tissues were dissected with scalpels and homogenized with a Polytron in guanidinium isothiocyanate buffer. Total RNA was purified with the help of the GeneElute Total RNA miniprep kit from Sigma-Aldrich Co. Purified RNA from tube feet was denatured at 65°C, cooled to room temperature and transcribed into first strand cDNA by reverse transcriptase from Moloney Murine Leukemia Virus (1.5U/μl) as suggested by the supplier of the enzyme (Stratagene). Random hexamers served as primers. Aliquots of this cDNA were amplified by PCR through 39 cycles (30 sec 95°C, 1 min 56°C, 1.5 min 68°C) utilizing Pfx polymerase (Invitrogen) and the primer pair GATAGAATGGCTCGGGTTTCGTC/AGCGTATGCTAGCAATGAAGAATG. The PCR product was resolved on a 1% agarose gel. The DNA band of 1600 bp was excised, purified on an Illustra GFX column (GE Healthcare Europe GmbH) and directly sequenced by the dideoxy chain termination method with a cycle sequencing machine (ABI 3730). For subcloning, the PCR band was re-amplified as above with the primer pair AAGAATTCATGGCTCGGGTTTCGT/AGCTCGAGCTAGCAATGAAGAATG. The product was digested with Eco RI and Xho I and the resulting two bands were ligated into the sequencing vector pBluescript SK(-).

### Bioinformatics

All sequences were analyzed with the GCG computer software package of Accelrys (Cambridge, UK). Similarities were calculated with the program Old Distances using the Blosum 62 scoring matrix. A multiple sequence alignment was performed with the program Pileup. For phylogenetic analysis, only the extracellular domains without signal peptides were used. An unrooted tree was built by the neighbour joining method using the program PaupSearch. Bootstrap values were calculated for all nodes from 1000 random replicates [17].

### Northern Blot

Total RNA from different tissues was separated on a 1% agarose gel in the presence of formaldehyde and transferred to a Nylon membrane by vacuum blotting [1,10]. The blot was hybridized overnight at 42°C with a radioactively labelled cDNA probe in a buffer containing 50% formamide. This probe had been labelled beforehand with [α-^32^P]-dCTP by the random primed oligolabelling method. After washing with standard saline citrate (SSC), the blot was exposed to an X-ray film.

### Fusion proteins

Three fragments derived from the intracellular domain of human and S. purpuratus FGFRL1 were prepared in a prokaryotic expression system as fusion proteins with glutathione S-transferase (GST). To this end, the cDNA sequences for human residues 400-471 (human up), human residues 472-504 (human low) and S. purpuratus residues 492-532 (sea urchin low) were subcloned into the Bam HI/Xho I restriction site of the expression vector pGEX-5X-2 (GE Healthcare) downstream of the GST gene. The resulting plasmids were transfected into competent bacteria (*E. coli *BL21). Fusion proteins were expressed after induction with isopropylthio-β-galactoside as suggested by the supplier of the GST gene fusion system (GE Healthcare). The bacteria were collected by centrifugation, resuspended in 50 mM Tris, pH 8.0, 150 mM NaCl, 1 mM phenylmethanesulfonyl fluoride, 0.3 mM ZnCl_2_, 1% Triton X-100 and lysed by sonication. Fusion proteins were purified from the lysates by affinity chromatography on GSH Sepharose [25] according to the instructions of the supplier (GE Healthcare). The purified proteins were dialyzed at 4°C against 50 mM Tris, pH 8.0, 150 mM NaCl, 1% Triton X-100 (4 changes over a total of 4 days). In order to remove any traces of metal ions, the dialysis buffer had been passed beforehand over a column (1 cm × 10 cm) of Chelex A100 (BioRad).

### Western blots

Proteins were analyzed by SDS polyacrylamide gel electrophoresis on gels containing a 5% stacking and a 15% running gel. After transfer to nitrocellulose by electroblotting, the polypeptides were detected with the GST detection module (GE Healthcare) using goat anti-GST antibodies, followed by alkaline-phosphatase conjugated secondary antibodies (Sigma). The colour reaction was performed with bromochloroindolyl phosphate and nitroblue tetrazolium as substrate.

### Affinity chromatography

Recombinant proteins dissolved at 70 μg/ml in phosphate buffer (50 mM sodium phosphate, pH 8.0, 300 mM NaCl, 20 mM imidazole, 1% Triton X-100) were mixed with pre-equilibrated His-Select™ Nickel affinity beads (Sigma). The suspension was incubated for 30 min at room temperature, before the beads were washed with the same buffer. Specifically bound proteins were eluted with 1.2 M imidazole, 50 mM sodium phosphate, pH 8.0, 300 mM NaCl, 1% Triton X-100 and analyzed by Western blotting.

To prepare zinc affinity columns, the His-Select™ Nickel affinity gel was treated with 100 mM EDTA, followed by extensive washing and re-loading with 40 mM zinc chloride. The exchange of the nickel ions could be detected by a specific colour change of the gel from light blue to white.

### Atomic absorption

The determination of the zinc and copper content of the fusion proteins was performed in triplicates using a SpectrAA-110 flame atomic absorption spectrophotometer (Varian Corp., Australia). Prior to metal analysis, the samples were diluted into 15 mM HNO_3 _matrix solution. Zn- and Cu-standards were prepared from commercially available 1000 ppm standards (Fluka, Buchs, Switzerland) using the same matrix solutions.

## Abbreviations

FGF: fibroblast growth factor; FGFR: fibroblast growth factor receptor; FGFRL1: fibroblast growth factor receptor-like 1; GST: glutathione S-transferase.

## Authors' contributions

LZ and BT designed the study, LZ carried out the biochemical experiments, AVK performed atomic absorption spectroscopy, PB collected the animals, BT drafted the manuscript. All authors read and approved the final paper.
